# Variation in rates of spontaneous male production within the nematode species *Pristionchus pacificus* supports an adaptive role for males and outcrossing

**DOI:** 10.1186/s12862-017-0873-7

**Published:** 2017-02-23

**Authors:** Katy Morgan, Angela McGaughran, Christian Rödelsperger, Ralf J. Sommer

**Affiliations:** 1 Department for Evolutionary Biology, Max Planck Institute for Developmental Biology, Tübingen, 72076 Germany; 2 Department of Biological Sciences, University of New Orleans, 2000 Lakeshore Drive, New Orleans, LA70148 USA; 3 CSIRO Land & Water, Black Mountain Laboratories, Clunies Ross Street, Canberra, ACT 2601 Australia; 4 University of Melbourne, School of BioSciences, 30 Flemington Road, Melbourne, VIC 3010 Australia

**Keywords:** Androdioecy, Outcrossing, Nematode, *Pristionchus pacificus*, Linkage disequilibrium

## Abstract

**Background:**

The nematode species *Pristionchus pacificus* has an androdioecious mating system in which populations consist of self-fertilizing hermaphrodites and relatively few males. The prevalence of males in such a system is likely to depend on the relative pros and cons of outcrossing. While outcrossing generates novel allelic combinations and can therefore increase adaptive potential, it may also disrupt the potentially beneficial consequences of repeated generations of selfing. These include purging of deleterious alleles, inheritance of co-adapted allele complexes, improved hermaphrodite fitness and increased population growth. Here we use experimental and population genetic approaches to test hypotheses relating to male production and outcrossing in laboratory and natural populations of *P. pacificus* sampled from the volcanic island of La Réunion.

**Results:**

We find a significant interaction between sampling locality and temperature treatment influencing rates of spontaneous male production in the laboratory. While strains isolated at higher altitude, cooler localities produce a higher proportion of male offspring at 25 °C relative to 20 or 15 °C, the reverse pattern is seen in strains isolated from warmer, low altitude localities. Linkage disequilibrium extends across long physical distances, but fails to approach levels reported for the partially selfing nematode species *Caenorhabditis elegans*. Finally, we find evidence for admixture between divergent genetic lineages.

**Conclusions:**

Elevated rates of laboratory male generation appear to occur under environmental conditions which differ from those experienced by populations in nature. Such elevated male generation may result in higher outcrossing rates, hence driving increased effective recombination and the creation of potentially adaptive novel allelic combinations. Patterns of linkage disequilibrium decay support selfing as the predominant reproductive strategy in *P. pacificus.* Finally, despite the potential for outcrossing depression, our results suggest admixture has occurred between distinct genetic lineages since their independent colonization of the island, suggesting outcrossing depression may not be uniform in this species.

**Electronic supplementary material:**

The online version of this article (doi:10.1186/s12862-017-0873-7) contains supplementary material, which is available to authorized users.

## Background

Androdioecy, a rare mating system in which populations are comprised of both hermaphrodites and males, has evolved multiple times in several genera of rhabditid nematodes, e.g. [1–3], reviewed in [4]. Their ability to both produce and fertilize eggs gives hermaphrodites a distinct advantage over males. In nematode species such as *Pristionchus pacificus* and *Caenorhabditis elegans,* hermaphrodites are able to self-fertilize and hence reproduce without suffering physiological costs associated with copulation and mate searching. The ability to self-fertilize confers the advantage of reproductive assurance in situations where population density is low and potential mates scarce, such as during long-distance dispersal and colonization [2–6]. Indeed, a selfing strategy has been shown to provide reproductive assurance within experimental *C. elegans* populations exposed to novel environmental conditions in which outcrossing is limiting [5]. The reduced time-cost of reproduction resulting from self-fertilization (as time is no longer required to seek mates) is proposed to have led to weakened selection for longevity, resulting in reduced lifespan in androdiocious relative to dioecious *Pristionchus* species [7]. The evolutionary advantage of hermaphrodites begs the question of why males persist within natural populations of such androdioecious species. Since hermaphrodites are only capable of self-fertilization, the impact of males will depend on the advantages and disadvantages of selfing versus outcrossing.

Theory predicts repeated generations of selfing will result in the purging of deleterious alleles and consequent reduction of inbreeding depression (e.g. [8–11]), reviewed in [12]. Indeed, experimental evolution studies have found support for reduced inbreeding depression in androdioecious relative to dioecious *Caenorhabditis* populations, with inbred androdioecious populations showing higher survival rates than their inbred dioecious counterparts [13, 14]. Elevated linkage disequilibrium in populations with a high frequency of selfing is likely to lead to the evolution of gene complexes, either through selective sweeps or genetic drift. This accelerates the rate of lineage divergence and increases the likelihood of outcrossing depression, as effective recombination may break up those genomic combinations which are adaptive. Outcrossing depression has both been inferred in natural *C. elegans* populations [15] and demonstrated in laboratory populations, in which crosses between genotypes from geographically distinct regions result in particularly strong reductions in F1 fitness [12]. Such reduced hybrid fitness has also been documented following crosses between geographically disparate strains of *P. pacificus* [16–18].

The persistence of males in an androdioecious system thus has the potential to not only slow population growth and reduce hermaphrodite fitness, but also to cause outcrossing depression (reviewed in [4]). It has been proposed that male persistence at low frequencies has no selective advantage in androdioecious nematode species and is rather due to a constant low level of mutation leading to spontaneous male generation [3]. However, experimental evolution studies within *C. elegans* suggest some evolutionary advantage of outcrossing may be conferred through selection favoring novel recombinants and overdominant loci in androdioecious populations [13]. Generations of selfing can cause advantageous alleles to remain linked to genetic backgrounds that have overall lower fitness, thus limiting their adaptive potential. The effective recombination resulting from outcrossing generates novel genomic combinations, thus increasing adaptive variation, while outcrossing can also help maintain diversity at overdominant loci [13]. Outcrossing is therefore especially likely to confer selective advantages during exposure to novel and/or stressful environmental conditions. In support of this theory, selection on male performance and outcrossing has been demonstrated in experimental *C. elegans* populations exposed to novel environments [19]. In addition, elevated outcrossing rates have been found to enable laboratory *C. elegans* populations to adapt to pathogenic conditions where obligate selfing populations were unable to persist [20], while increased outcrossing in experimental *C. elegans* populations lacking initial genetic diversity was found to correlate with increasing fitness levels [3].

*Caenorhabditis elegans* and *Pristionchus pacificus* are distantly related nematode species, and despite their similar reproductive strategies are characterized by very different evolutionary histories. While *C. elegans* populations exhibit low genetic diversity and a lack of geographic structure even across continents [21, 22], *P. pacificus* populations are characterized by high genetic diversity and strong fine-scale geographic structure [23–26]. These differences may in part reflect differences in outcrossing rates between species, yet although rates of male generation and putative adaptive roles for outcrossing have been extensively studied in laboratory populations of *C. elegans,* the topic has been little explored in *P. pacificus.*

**Fig. 1:**
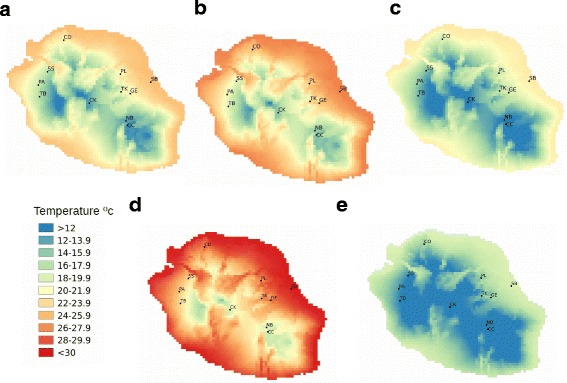
Map of La Réunion Island, indicating the following climatic variables experienced at each of the 11 sampling localities: **a**. mean annual temperature; **b**. mean temperature of the warmest quarter; **c**. mean temperature of the coolest quarter; **d**. maximum temperature of the warmest month; **e**. minimum temperature of the coolest month. Climate data has been downloaded from WorldClim

Additionally, although natural variation in rates of male generation has been demonstrated in both *P. pacificus* and *C. elegans* [15, 27], it is not known how this relates to variation in the adaptive history of natural populations encountering different environmental conditions. Volcanic islands are the ideal sites to test theories relating to adaptive processes, as land-based flora and fauna must have arrived by migration before experiencing local adaptation in isolation from mainland populations. The island of La Réunion is the youngest of the Mascareigne islands, a volcanic island chain in the Indian Ocean. Just 2-3 million years old, the island has an active volcano and a consequent rugged topography, characterized by substantial environmental changes over short geographic distances [28]. As can be seen from Fig. 1, temperature profiles vary dramatically across the island. Whereas sea-level localities are generally characterized by a mean annual temperature of 24-26 °C and a minimum temperature of approximately 18 °C, high-altitude localities in the center of the island experience mean annual temperatures of 14-16 °C and maximum temperatures not exceeding 20 °C. *P. pacificus* is common on the island and distributed from sea-level to high-altitude montane plateaus [24, 29], hence typical daily temperature is likely to vary considerably between populations.

The presence of four distinct genetic lineages of *P. pacificus* on La Réunion Island has been previously detected and characterized through Maximum-Likelihood phylogenetic analysis of mitochondrial DNA [23], bayesian clustering analysis of nuclear microsatellites [24] and principal components analysis of genome-wide SNP data [25]. These lineages are demonstrated to have arisen from several independent colonizations of the island from geographically disparate source populations [30]. Details of the localities at which each lineage has been detected is illustrated in Additional File 1. Two lineages, designated C and D, respectively [23–25], have been sampled over a wide and overlapping distribution, with C being more common on the arid south-western side of the island and D being more common in the humid north-east. The distributions of lineages A and B appear to be confined primarily to low-altitude localities in the north-east and to high-altitude, cool montane plateau regions, respectively (Additional File 1) [23–25]. Strains isolated from a large number of La Réunion localities and ecozones for these studies have been maintained as frozen cultures in the laboratory, and are available for experimental and ecological studies.

Here we exploit the advantages of the *P. pacificus* system to test predictions relating to the potential adaptive role of males and outcrossing. We use a subset of 120 laboratory-maintained strains covering all four genetic lineages and 11 sampling localities (see Fig. 1 and Table 1) across La Réunion Island to determine how rates of male production vary according to sampling locality and temperature treatment. We predict that if male production and outcrossing are adaptive responses to novel and/or stressful conditions, both the climate experienced within the natural population and the temperature experienced in the laboratory will influence the rate of spontaneous male generation. We also aim to test the prediction of elevated linkage disequilibrium within each of the distinct genetic lineages of *P. pacificus* on La Réunion Island, comparing patterns observed to those reported for other partially selfing species including *C. elegans.* If outcrossing is rare within natural populations, we expect to see linkage disequilibrium extending over long genetic distances in all lineages. Finally, we aim to characterize patterns of fine-scale population structure in *P. pacificus* on La Reunion Island, specifically to determine whether data supports admixture between distinct genetic lineages. If outcrossing depression is typically strong within natural populations, we would expect a complete lack of admixture between distinct genetic lineages in regions where their ranges overlap.

**Table 1:** Sampling localities and number of strains included

Sampling locality	GPS position	Number of strains
Le Cratere Commerson (CC)	-21.3203N, 55.71065E	9
Coteau Kerveguen (CK)	-21.2074N, 55.64383E	7
Colorado (CO)	-21.1300N, 55.5123E	10
Grand Etang (GE)	-21.09687N, 55.6535E	10
Neu du Boeuf-Vulcano (NB)	-21.1919N, 55.63945E	7
Des Palmistes (PA)	-21.06696N, 55.33474E	6
Plaines des Lianes (PL)	-21.02845N, 55.61872E	9
Saint Benoit (SB)	-21.05728N, 55.72543E	22
Sans Souci (SS)	-21.0205N, 55.36697E	10
Trois Bassin (TB)	-21.1083N, 55.33865E	30
Takamanda (TK)	-21.09084N, 55.62128E	6

## Methods

### Sample selection

*Pristionchus pacificus* adults can be found either loose in the soil or on the bodies of scarab beetles, with which they have a necromenic association, resting on the beetle in a state of arrested development before emerging to feed on the beetle carcass once it dies. Isogenic lines have been established for previous studies [23, 24, 31] by sampling single hermaphrodites from natural populations, allowing them to reproduce via self-fertilization and maintaining their offspring in culture. Each isogenic line represents a single strain.

We first aimed to estimate rates of spontaneous male production in a subset of 120 strains isolated from 11 sampling localities (Fig. 1, Table 1) across La Réunion Island, under three temperature treatments (15 °C, 20 °C and 25 °C). These temperatures are expected to represent typical temperatures for high, mid-elevation and sea-level populations, respectively, based on climatic data available from WorldClim (Fig. 1). Wild populations were sampled during January of each of the years 2010, 2011 and 2012. A cluster of strains isolated from similar sampling points (within a radius of approximately 500m) is considered to represent a population. Established cultures of all strains were frozen at -80 °C until the start of the experiment. Since development and therefore reproduction ceases at -80 °C, adaptation to laboratory conditions through novel mutations or changes in the frequency of existing alleles is expected to be minimal.

### Estimation of rates of spontaneous male production

Each strain was thawed at room temperature prior to the assay and split into three cultures, maintained at each of 15 °C, 20 °C and 25 °C. Cultures were maintained at the temperature of the assay for four generations prior to the start of the assay. For each strain, ten J4 virgin hermaphrodites were transferred to a 10 cm diameter NGM agar plate seeded with 200 μl of *Escherichia coli* strain OP50. Once the hermaphrodites reached maturity and began laying eggs, they were transferred daily to a freshly seeded plate. This ensured all offspring on a single plate were at approximately the same stage of development, and that adult hermaphrodites couldn't mate with any male offspring. Daily transfer of the hermaphrodites was continued until they were no longer laying fertilized eggs. Once offspring on a given plate reached maturity the numbers of males and hermaphrodites were counted using clicker counters. Plates were frozen immediately prior to counting to reduce the movement of individual nematodes. Male counts were recorded as the exact number of individual males on each plate. Since hermaphrodites were often clumped into groups of individuals and it was difficult to determine the exact number of individuals within each clump, hermaphrodite counts were determined as closely as possible and recorded to the nearest 50 individuals. Counts were pooled across all offspring for a given set of ten hermaphrodites to give the number and estimated percentage of males produced for each strain. Three replicates of this protocol were performed for each strain, providing three sets of count data for each strain under each temperature treatment.

### Statistical analyses

Generalized linear mixed models were performed using the R package LME4v1.1-8 [32] to determine the influence of experimental temperature treatment and sampling locality on the percentage of male offspring. Temperature treatment and sampling locality were defined as fixed effects and strain was included as a nested random effect. The interaction between temperature treatment and sampling locality was included in the model. Specificities of the interaction between temperature treatment and sampling locality were examined further using post-hoc Tukey's tests.

To examine the data further, sampling localities were pooled into “cold” (NB, CC, CK), “mid-cold” (PA, TB, SS), ”mid-warm” (CO, TK, PL, GE) and “warm” (SB) climate groups according to the climate data available from WorldClim (Fig. 1). A second generalized linear model was performed to determine the influence of temperature treatment and sampling locality climate group on the percentage of males produced. Temperature treatment and climate group and their interaction were included as fixed effects and sampling locality and strain were included as random nested effects. Again, specificities of the interaction between temperature treatment and climate group were examined further using post-hoc Tukey's tests. All analyses were conducted using the statistical package R [33].

### Estimation of recombination rates and the decay of LD

We aimed to test the prediction of linkage disequilibrium extending across long physical distances in all four distinct lineages, indicative of low levels of outcrossing. Analyses were conducted using the SNP dataset generated in [25, 26] (deposited in the European Nucleotide Archive, PRJEB13695), consisting of 29,810 SNPS from 6 chromosomes and 251 individuals. We used Haploview [34] to calculate squared allele frequency correlations, r^2^ [35], between pairs of polymorphic sites within the longest 30 assembled contigs within each of the four genetic lineages. The decay of LD over distance was characterized through non-linear regressions of LD between sites, estimated as r^2^, vs. the distance between sites in base pairs [36]. The following equation describes the expected decay of r^2^ over distance [37]:

$$ E\left({r}^2\right)=\left[10 + C/\left(2 + C\right)\left(11 + C\right)\right] \times \left[1+\left(\left(3 + C\right)\left(12 + 12C + {C}^2\right)/\ n\left(2 + C\right)\left(11 + C\right)\right)\right] $$

where n is the sample size and C represents the product of the population scaled mutation rate (ρ = N_e_r) and the distance in base pairs. This equation was fitted using the R statistical package [33] and used to determine the distance over which r^2^ decays to half its original value.

### Inference of population structure and admixture

Finally, we aimed to characterize fine-scale patterns of population structure and infer whether admixture has occurred between the four distinct genetic lineages since their independent colonization of the island. In the presence of strong outcrossing depression, outcrossing between divergent genetic lineages in regions of secondary contact is expected to be negligible. Analyses were performed within the ChromoPainter pipeline [38], which has been demonstrated to be effective in detecting fine-scale population structure and inferring admixture within contemporary populations (e.g. [39, 40]). Briefly, for each locus on each individual chromosome there exists within the total genetic dataset a closest relative. The locus and the closest relative are inferred to have been co-inherited from a common ancestor. Loci that are closer together on a chromosome are more likely to be physically linked and therefore to have been co-inherited from the same common ancestor, hence 'chunks' of haplotypes are inferred to have been co-inherited between pairs of individuals [38]. ChromoPainter uses a Hidden Markov Model (HMM) to reconstruct every haplotype as 'chunks' which are 'donated by' (this is analogous to being co-inherited from a recent common ancestor) haplotypes of other individuals in the dataset. This information is used to generate a coancestry matrix, which indicates the relationship between pairs of individuals across all sampled regions of the genome. The program fineSTRUCTURE is then used to implement a model-based Bayesian clustering algorithm to infer relationships and similarities between populations, using the coancestry matrix produced in ChromoPainter.

This pipeline is suitable for large SNP datasets, and a particular advantage is that the recombination distance between pairs of SNP loci is taken into account, such that SNP pairs that are physically closer together on the chromosome are assumed to be more likely to be inherited together [38]. This provides an advantage over other clustering algorithms and is especially pertinent for a partially selfing species such as *P. pacificus*.

Using the same SNP dataset as the linkage and recombination analysis, Chromopainter ver. 2 [38] was run using the default settings and ten Expectation-Maximisation (EM) replicates. The -a 0 0 option was used, facilitating the simultaneous analysis of all individuals such that all haplotypes in the dataset could be considered potential 'donors' (or closest neighbours) to each reconstructed haplotype. FineSTRUCTURE [38] was then used to confirm convergence of ChromoPainter runs, perform an MCMC iteration on the co-ancestry matrix and create an aggregated matrix illustrating the relationships between inferred clusters of haplotypes. This was performed on the 'chunkcounts' ChromoPainter output, which represents the number of chunks each haplotype co-inherits from each of the other haplotypes in the dataset. Analyses were performed using the linked model, which does not assume independence of loci and takes into account recombination distances between pairs of loci, using a burnin and runtime of 500,000 and 5,000,000 iterations, respectively, and 10,000 MCMC samples.

## Results

### Prediction 1: male generation can be predicted by an interaction between sampling locality climate and temperature treatment

All male and hermaphrodite count data can be found in Additional File 2. The GLM and statistically significant post-hoc Tukey's comparisons are shown in Table 2 and Table 3, respectively. While temperature treatment and sampling locality were found not to have a significant influence on the percentage of males produced (df = 1 and 10, *F* = 0.939 and 0.547, *p* = 0.335 and 0.853, respectively), a significant interaction between temperature treatment and sampling locality was detected (df = 3, *F* = 4.201, *P* = 0.007; see Table 2). The post-hoc Tukey's test revealed a significant influence of temperature treatment on the percentage of males produced by strains sampled from the following four sampling sites (see Table 3): CC and NB, with strains producing significantly higher percentages of males at 25 °C relative to both 20 °C and 15 °C (CC: *z* = 3.941 and 4.442, *p* = 0.02 and <0.01, respectively; NB: *z* = 3.785 and 5.877, *p* = 0.036 and <0.01, respectively); CO and GE, with strains producing significantly higher percentages of males at 15 °C relative to 25 °C (CO: *z* = 5.711, *p* < 0.01; GE: *z* = 4.616, *p* < 0.01). Hence at two out of three of the sampling localities with a “cold” climate (see Fig. 1), significantly higher percentages of males were produced at the highest relative to the lowest temperature treatment, whereas at two of the four sampling localities with a “mid-warm” climate, significantly higher percentages of males were produced at the lowest relative to the highest temperature treatment. It is possible that small sample sizes and the tendency for some strains to fail to propogate at putatively stressful temperatures (which effectively reduces the sample size even further) may be responsible for the lack of significant effects at other sample sites. We aimed to reduce this effect by combining sampling sites by climate group (see Fig. 1).

**Table 2:** Statistical results for GLMs testing the influence of a. temperature treatment and sampling locality on the percentage of male offspring; and b. temperature treatment and sampling locality climate group on the percentage of male offspring

a.			
Factor	Df	*F* value	*P*-value
Temperature	1	1.104	0.296
Sampling Locality	10	1.817	0.067
Temperature: Sampling Locality	5	4.918	<0.001 ***
b.			
Factor	Df	*F* value	*P* value
Temperature	1	0.977	0.325
Climate Group	3	0.751	0.524
Temperature: Climate Group	2	4.918	0.002 **

In a. temperature treatment, sampling locality and their interaction are defined as fixed effects and strain is included as a nested random effect. In b. temperature treatment, sampling locality climate group and their interaction are defined as fixed effects and strain is included as a nested random effect. Significant effects are indicated by asterisks: *, ** and *** indicate significance at the *p* < 0.05, *p* < 0.01 and *p* < 0.001 level

**Table 3:** Post-hoc Tukey's test results showing significant effects of a. sampling locality and temperature treatment, and b. sampling locality climate group and temperature, on the percentage of males produced

a.		
Sampling locality and treatment pair	Z-value	*P*-value
CC 25 - CC 15 == 0	4.442	<0.01 **
CC 25 - CC 20 == 0	3.941	0.020 *
CO 25 – CO 15 == 0	- 5.711	<0.01 **
GE 25 – GE 15 == 0	- 4.616	<0.01 **
NB 25 – NB 15 == 0	5.877	<0.01 **
NB 25 – NB 20 == 0	3.785	0.036 *
b.		
Climate group and treatment pair	Z-value	*P*-value
cold 25 - cold 15 == 0	6.468	<0.001 ***
cold 25 - cold 20 == 0	5.309	<0.001 ***
mid-cool 20 – mid-cool 15 == 0	- 3.235	0.042 *
mid-warm 25 – mid-warm 20 == 0	- 3.785	0.006 **
mid-warm 20 – mid-warm 15 ==0	- 3.612	0.012 *
mid-warm 25 – mid-warm 15 == 0	- 7.397	<0.001 **
Midhigh 25 – High 25 == 0	- 3.890	0.004 **
Midlow 25 – High 25 == 0	- 4.200	0.001 **

Significant effects are indicated by asterisks: *, ** and *** indicate significance at the *p* < 0.05, *p* < 0.01 and *p* < 0.001 level

**Fig. 2:**
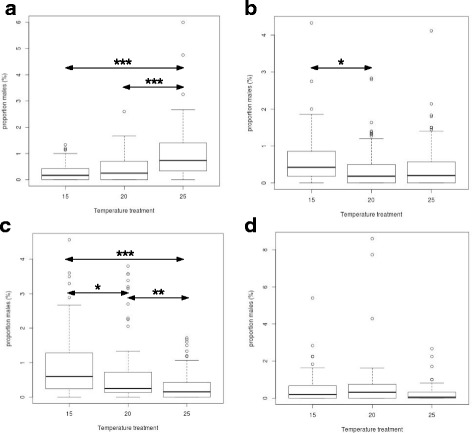
Box-and-whisker plots displaying the median (solid line within each box), upper and lower quartiles (upper and lower extent of each box, respectively), upper and lower extremes ('whiskers' extending from each box) and outlier values for the percentage of males produced under each experimental group. An experimental group corresponds to strains isolated from sampling localities grouped within a specific climate group, exposed to a specific temperature treatment. Climate groups are defined according to the predominant climate experienced at the specific sampling locality. Within each experimental group, each strain is represented by three replicates. Significant differences between experimental groups in the percentage of males produced are indicated by asterisks: *, ** and *** indicate significance at the *p* < 0.05, *p* < 0.01 and *p* < 0.001 level. Box-and-whisker plots are displayed for strains grouped according to the following: **a**. cold climate sampling localities (CC, CK and NB); **b**. mid-cold climate sampling localities (TB, PA and SS); **c**. mid-warm climate sampling localities (CO, GE, PL and TK); **d**. warm climate sampling localities (SB). Whereas significantly higher proportions of males are produced under 25 °C relative to 20 °C and 15 °C within the “cold” climate group (plot a), this pattern is significantly reversed in the “mid-warm” climate group (plot c)

When sampling localities were grouped into climate groups, the interaction between temperature treatment and climate group was found to be significant (df = 2, *F* = 6.53, *P* = 0.002; see Table 2), while the independent effects of temperature treatment and climate group were non-significant (df = 1 and 3, *F* = 2.172 and 1.668, *p* = 0.325 and 0.524, respectively). Post-hoc Tukey's tests are again shown in Table 3, and reveal significant temperature treatment effects within the “cold”, “mid-cool” and “mid-warm” climate groups. Strains from the “cold” climate group produced significantly higher proportions of males at 25 °C relative to 20 °C and 15 °C (*z* = 5.309 and 6.468, respectively, *p* < 0.001); strains from the “mid-cold” climate group produced significantly higher proportions of males at 15 °C relative to 20 °C (*z* = 3.235, *p* = 0.042); and strains from the “mid-warm” climate group produced significantly higher proportions of males at 15 °C relative to both 20 °C and 25 °C (*z* = 3.612 and 7.397; *p* = 0.012 and <0.001, respectively), and higher proportions of males at 20 °C relative to 25 °C (*z* = 3.785, *p* = 0.006). Thus the pattern of producing higher proportions of males at higher temperatures, as detected in the “cold” climate group, is reversed in the “mid-warm” climate group, as can be seen in the boxplots illustrated in Fig. 2. The lack of significant temperature treatment effects within the “warm” climate group may partly reflect the fact that many strains within this group failed to propogate at 15 °C.

### Prediction 2: Linkage disequilibrium extends across long physical distances in all four genetic lineages

**Fig. 3:**
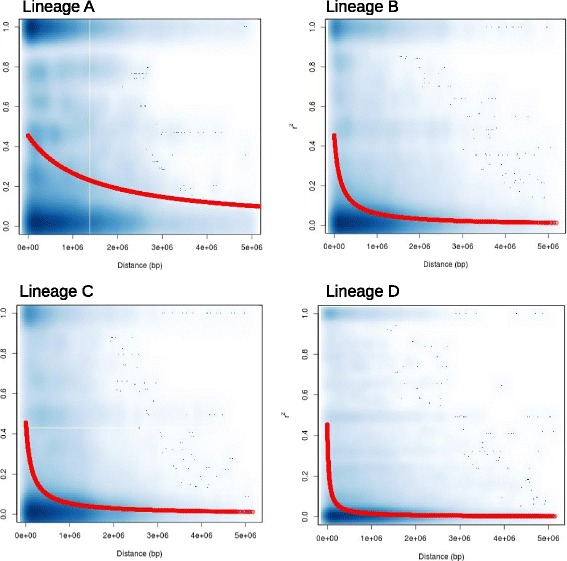
Scatter plots illustrating the decay of linkage disequilibrium within each of the four genetic lineages, as estimated using r^2^, over physical distance. The r^2^ values between each pair of sites is shown in blue, smoothed using the smoothScatter function in R. The red line illustrates the nonlinear regression of r^2^ over physical distance, as determined using the method described in [32]. R was found to reach half of its original value in a physical distance of 1.42Mb, 150kb, 130kb and 33kb within lineages A, B, C and D, respectively

Linkage disequilibrium was measured by calculating r^2^ between pairs of SNPs and fitting a function (see Methods) to quantify the decay of LD with increasing physical distances (Fig. 3). LD was found to extend across large physical/genomic distances within all four lineages (see Fig. 3), with r^2^ declining to approximately half of the minimum value within 1.421Mb, 0.147Mb, 0.131Mb and 33kb in lineages A, B, C and D respectively.

### Prediction 3: A lack of admixture detected between distinct genetic lineages

**Fig. 4:**
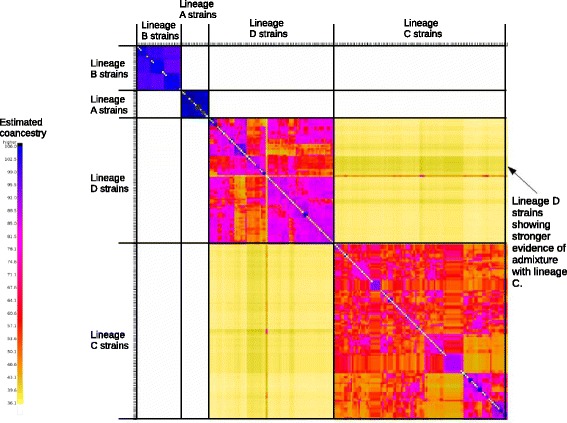
Coancestry heat-map indicating relationships between strains, as generated using Chromopainter and FINEstructure. Each row and each column represents the estimated coancestry between each individual strain and each of the other strains in the dataset. Strains are grouped by lineage, as indicated. Coancestry ranges from 0 (white) to 1 (black); the darker the colour the higher the estimated coancestry. Individuals within lineage *A* and *B* show high coancestry with strains from the same genetic lineage and zero coancestry with individuals from other genetic lineages. Lineages *C* and *D* show highest coancestry with individuals from the same genetic lineage, however admixture between lineages *C* and *D* is supported by the moderate levels of coancestry between lineage *C* and *D* individuals

The aggregated heat-map in Fig. 4 shows the inferred coancestry between pairs of individuals. Each row and column represents the comparison of one individual with all other individuals in the dataset. Individuals are sorted by genetic lineage, as indicated. The darker the colour, the higher the degree of shared ancestry inferred. Individuals within lineage A and B show a high degree of coancestry with other individuals from the same genetic lineage, as indicated by the dark blue and purple coancestry blocks, and a low or negligible degree of coancestry with individuals from other genetic lineages. This supports a lack of recent admixture between lineages A, B and all other lineages.

Individuals within lineages C and D, however, show a higher degree of coancestry with one another, as evidenced by the yellow heat-map colors between pairs of individuals from the alternate lineage. One lineage D group shows a particularly high proportion of shared ancestry with C haplotypes (as indicated on Fig. 4) suggesting this group has resulted from admixture between the two lineages. This group includes strains isolated from two localities: GE and BV, where the distribution of lineages C and D has been found to overlap.

## Discussion

### Effects of sampling locality and temperature treatment on proportion of male offspring

We demonstrate significant interactions between temperature treatment and both sampling locality and sampling locality climate group on rates of male production in laboratory populations of *P. pacificus*. This supports previous analyses indicating temperature treatment in the laboratory has a significant effect on levels of spontaneous male generation, and that the effect of temperature treatment differs between strains [27]. By analyzing a larger number of strains isolated from sampling localities that differ in climate, we add an environmental context.

Strains isolated from montane localities within the “cold” climate group, at which maximum temperatures remain below 18 °C, show significantly elevated rates of male production under maintenance at 25 °C relative to either 20 °C or 15 °C. Strains isolated from high-elevation localities within the “mid-cool” climate group, at which temperatures range from 14 °C to 26 °C, show a more even rate of male generation across temperature treatments. Meanwhile, strains isolated from the mid-to-low elevation, “mid-warm" climate group localities, at which minimum temperatures remain above 18 °C, produce significantly higher proportions of males under 15 °C relative to 20 °C and 25 °C. Thus male production tends to be elevated under temperatures strains are unlikely to experience in their natural environment.

This suggests elevated male generation may be a stress response, triggered by exposure to temperatures outside of the range typically experienced by natural populations. These findings are supported by experimental evolution studies in *C. elegans*, which demonstrate elevated rates of male generation and outcrossing in laboratory populations subjected to environmental stresses including starvation [20] and exposure to a novel and virulent bacterial pathogen [15, 41]. Such adaptive advantages of periodic outcrossing may be conferred through selection on novel recombinants and overdominant loci [13]. Although natural variation amongst strains in rates of male production has been demonstrated in *P. pacificus* as well as *C. elegans* (e.g. [15, 27]), this is the first documented support for increased male generation in response to environmental stress in *P. pacificus*, and to our knowledge, the first demonstration in androdioecious nematode populations of a link between sampling locality and rates of male production in the laboratory.

Rates of outcrossing in natural populations will be influenced by not only the rate of male generation, but also by the fitness of males produced. Experimental coevolution between *C. elegans* and a parasitic pathogen suggested the relative fitness of outcrossed offspring was more influential in maintaining outcrossing rates throughout exposure, since male fitness was in fact reduced by the pathogen presence [42]. However, increased male performance and selection on outcrossing throughout the experimental evolution of *C. elegans* populations exposed to non-pathogenic environments suggests male mating ability can also drive the maintenance of outcrossing [19]. While similar estimation of male fitness under environmental stress was outside the scope of this study, this should be a focus of future work.

### Levels of linkage disequilibrium

In keeping with predictions for partially-selfing species with low levels of outcrossing, LD decays slowly in all four genetic lineages. Whereas LD-decay within populations of gonochoristic *Caenorhabditis* species such as *C. remanei* occurs over hundreds of base pairs [43], equivalent LD-decay within distinct lineages of *P. pacificus* is estimated to be several orders of magnitude slower. Indeed LD-decay within lineage D (33kb) occurs over a comparable distance to those generally reported for partially-selfing plants such as legume, soybean and rice species and *Arabidopsis thaliana,* in which LD typically decays over 1-50kb [44–46].

Linkage disequilibrium detected within this study fails to approach levels reported in *C. elegans,* with which *P. pacificus* shares an androdiocious, partial selfing life history. Whereas LD in *C. elegans* decays over approximately 3.3Mb [47], decay is at least an order of magnitude faster in *P. pacificus* lineages B, C and D, occuring over 150, 130 and 33kb, respectively. This is consistent with higher levels of genetic diversity and structure reported within *P. pacificus* relative to *C. elegans*, suggesting relatively higher rates of outcrossing and/or effective population sizes within *P. pacificus*. The lower level of LD in *P. pacificus* with respect to *C. elegans* is consistent with findings from the partial selfer *C. briggsae* [48]. Studies in partially selfing species such as *Arabidopsis thaliana* show low but sustained levels of outcrossing are sufficient to generate and maintain diversity and structure within natural populations [49], hence even periodic outcrossing could be responsible for maintaining the considerable differences in population genetic patterns between *P. pacificus* and *C. elegans.*

Although it was not within the scope of this study to robustly quantify the differences in recombination across different lineages, we would like to point out that our data from *P. pacificus* shows considerable variation. The distance in which LD decays to half of the minimum value ranges between 33kb and 1.421Mb. The extensive LD detected within lineage A (decaying over 1.4Mb) is more comparable with levels observed in *C. elegans*. While this may reflect differential outcrossing rates and/or effective population sizes in lineage A, further study is required to rule out alternative explanations such as large-scale structural chromosomal changes in this lineage.

### Admixture between divergent lineages

Outcrossing depression formerly reported in *P. pacificus* (e.g. [15, 27]) would appear to contradict putatively elevated rates of outcrossing in this species relative to *C. elegans.* However, a comprehensive and rigorous analysis of outcrossing depression resulting from crosses between and within each of the *P. pacificus* genetic lineages, including strains which would have the opportunity to meet in nature, is thus far lacking. Indeed, our fineSTRUCTURE analysis provides support for admixture between lineages C and D on La Réunion Island and yet indicates a lack of admixture between lineages A and either C and D, despite their overlapping distributions. This suggests levels of outcrossing depression may depend on the nature of the cross, and may be less extensive within *P. pacificus* than previously thought. Periodic outcrossing between strains from divergent genetic lineages could generate high levels of structure and diversity, and may also contribute to the elevated genetic diversity, stronger genetic structure and less extensive LD within *P. pacificus* populations relative to *C. elegans.*

## Conclusions

Isolation of *P. pacificus* from a wide-variety of environments on the volcanic and ecologically heterogeneous island of La Réunion facilitates drawing links between conditions experienced in nature and the response of strains to various environmental pressures in the laboratory. Here we exploit this system to demonstrate elevated rates of male production under temperature regimes which likely differ from those experienced in nature, thus lending support to the hypothesis that male generation in androdioecious species such as *P. pacficus* is a stress response triggered by exposure to novel environmental conditions. Three of the four genetic lineages display rates of LD decay that are considerably higher than rates detected within the androdiocious species *C. elegans.* These patterns may reflect a history of higher outcrossing rates in *P. pacificus* relative to *C. elegans*, as well as admixture between certain genetic lineages, possibilities which warrant further research efforts.

## Additional files

Additional file 1: Sampling localities where each of the four genetic lineages have been previously detected on La Réunion Island. (PDF 1134 kb)
Additional file 2: Male and hermaphrodite count data recorded for each strain under each temperature treatment. (DOCX 19 kb)

## Abbreviations

SNP: Single nucleotide polymorphism
