# Supplementary material for: Variation in rates of spontaneous male production within the nematode species Pristionchus pacificus supports an adaptive role for males and outcrossing
Source: BMC Evol Biol. 2017 Feb 23;17:57. doi: 10.1186/s12862-017-0873-7 (PMC5322664; doi:10.1186/s12862-017-0873-7)
Supplement: Additional file 1: — Sampling localities where each of the four genetic lineages have been previously detected on La Réunion Island. (PDF 1134 kb) [file 12862_2017_873_MOESM1_ESM.pdf]

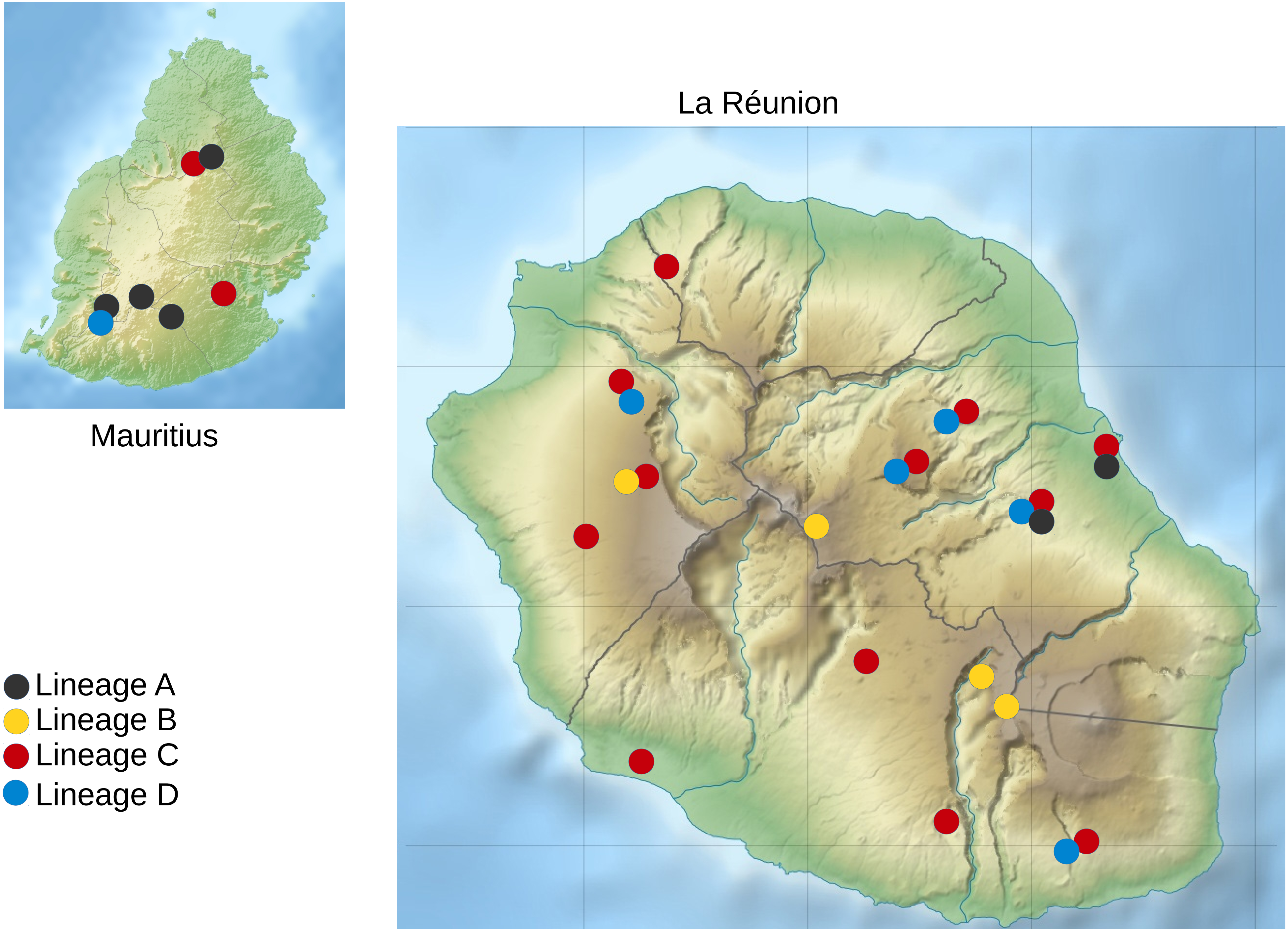

Additional File 1: Sampling localities where each of the four genetic lineages have been previously detected on La Réunion Island
