# Supplementary material for: Variation in rates of spontaneous male production within the nematode species Pristionchus pacificus supports an adaptive role for males and outcrossing
Source: BMC Evol Biol. 2017 Feb 23;17:57. doi: 10.1186/s12862-017-0873-7 (PMC5322664; doi:10.1186/s12862-017-0873-7)
Supplement: Additional file 2: — Male and hermaphrodite count data recorded for each strain under each temperature treatment. (DOCX 19 kb) [file 12862_2017_873_MOESM2_ESM.docx]

| Strain | Sampling locality | 15 °c temperature treatment | | | | | | 20ºc temperature | | | | | | 25ºc temperature | | | | | |
| --- | --- | --- | --- | --- | --- | --- | --- | --- | --- | --- | --- | --- | --- | --- | --- | --- | --- | --- | --- |
|  |  | Replicate 1 | | Replicate 2 | | Replicate 3 | | Replicate 1 | | Replicate 2 | | Replicate 3 | | Replicate 1 | | | Replicate 2 | | |
|  |  | Number males | Estimated hermaphrodites | Number males | Estimated hermaphrodites | Number males | Estimated hermaphrodites | Number males | Estimated hermaphrodites | Number males | Estimated hermaphrodites | Number males | Estimated hermaphrodites | Number males | Estimated hermaphrodites | Number males | Estimated hermaphrodites | Number males | Estimated hermaphrodites |
| RSB013 | CC | 2 | 500 | 3 | 500 | 4 | 500 | 0 | 300 | 0 | 400 | 0 | 400 | 3 | 600 | 4 | 400 | 2 | 400 |
| RSB001 | CC | 5 | 700 | 0 | 500 | 3 | 600 | 0 | 800 | 1 | 600 | 4 | 500 | 0 | 400 | 1 | 300 | 2 | 300 |
| RSB005 | CC | 6 | 500 | 1 | 600 | 2 | 600 | 6 | 600 | 1 | 600 | 4 | 400 | NA | NA | NA | NA | NA | NA |
| RSB008 | CC | 6 | 600 | 2 | 750 | 0 | 600 | 15 | 900 | 8 | 500 | 14 | 900 | 8 | 300 | 24 | 400 | 9 | 400 |
| RSC001 | CC | 3 | 650 | 1 | 700 | 5 | 600 | 2 | 700 | 5 | 1200 | 7 | 1000 | NA | NA | NA | NA | NA | NA |
| RSC002 | CC | 0 | 750 | 1 | 750 | 2 | 1000 | 2 | 700 | 6 | 1100 | 8 | 900 | NA | NA | NA | NA | NA | NA |
| RSC003 | CC | 0 | 400 | 1 | 750 | 0 | 600 | 0 | 750 | 1 | 500 | 0 | 400 | NA | NA | NA | NA | NA | NA |
| RSC004 | CC | 1 | 750 | 3 | 1000 | 4 | 700 | 0 | 500 | 2 | 700 | 2 | 1000 | NA | NA | NA | NA | NA | NA |
| RSC005 | CC | 0 | 700 | 0 | 1200 | 0 | 700 | 0 | 600 | 0 | 700 | 0 | 900 | NA | NA | NA | NA | NA | NA |
| RSC006 | CC | 0 | 700 | 0 | 1000 | 4 | 700 | 0 | 500 | 3 | 400 | 5 | 700 | NA | NA | NA | NA | NA | NA |
| RSC008 | CK | 0 | 500 | 1 | 700 | 2 | 900 | 1 | 800 | 3 | 1000 | 4 | 1000 | 0 | 500 | 2 | 800 | 1 | 400 |
| RSC010 | CK | 2 | 600 | 0 | 500 | 0 | 500 | 1 | 500 | 1 | 400 | 3 | 400 | NA | NA | NA | NA | NA | NA |
| RSC011 | CK | 4 | 500 | 3 | 850 | 2 | 500 | 1 | 700 | 0 | 800 | 1 | 400 | 1 | 700 | 5 | 600 | 4 | 800 |
| RSC012 | CK | 1 | 800 | 4 | 800 | 8 | 700 | 0 | 800 | 1 | 700 | 2 | 800 | 3 | 500 | 4 | 750 | 3 | 400 |
| RSC013 | CK | 0 | 500 | 0 | 1000 | 0 | 800 | 0 | 700 | 0 | 500 | 1 | 700 | 5 | 800 | 2 | 800 | 1 | 500 |
| RSC100 | CK | 4 | 500 | 3 | 700 | 3 | 750 | 0 | 500 | 1 | 600 | 3 | 650 | NA | NA | NA | NA | NA | NA |
| RSB020 | CO | 31 | 1500 | 25 | 1300 | 21 | 1400 | 0 | 400 | 1 | 600 | 4 | 500 | 1 | 900 | 3 | 800 | 3 | 900 |
| RSC014 | CO | 2 | 600 | 0 | 1000 | 6 | 900 | 1 | 750 | 0 | 900 | 4 | 900 | 1 | 1200 | 0 | 900 | 6 | 600 |
| RSC015 | CO | 5 | 800 | 21 | 1100 | 10 | 900 | 2 | 1000 | 11 | 1000 | 6 | 800 | 0 | 900 | 2 | 700 | 3 | 1350 |
| RSC016 | CO | 21 | 1000 | 3 | 900 | 8 | 800 | 1 | 400 | 0 | 800 | 0 | 700 | 2 | 600 | 1 | 750 | 0 | 600 |
| RSC017 | CO | 8 | 1150 | 10 | 1150 | 21 | 1150 | 3 | 600 | 1 | 800 | 2 | 1200 | 1 | 800 | 1 | 1000 | 0 | 500 |
| RSC018 | CO | 1 | 800 | 4 | 950 | 6 | 600 | 1 | 500 | 1 | 750 | 2 | 600 | 1 | 550 | 0 | 500 | 0 | 500 |
| RSC019 | CO | 2 | 600 | 2 | 1200 | 3 | 1250 | 2 | 600 | 0 | 600 | 0 | 600 | 0 | 500 | 0 | 600 | 0 | 600 |
| RSC020 | CO | 0 | 500 | 1 | 600 | 1 | 600 | 1 | 600 | 2 | 700 | 3 | 600 | 1 | 700 | 2 | 900 | 1 | 750 |
| RSC021 | CO | 21 | 1700 | 39 | 1350 | 73 | 1600 | 38 | 1850 | 44 | 1300 | 50 | 1400 | 3 | 500 | 1 | 550 | 1 | 1050 |
| RSC022 | CO | 9 | 800 | 7 | 1250 | 1 | 600 | 1 | 500 | 2 | 600 | 1 | 500 | 0 | 500 | 3 | 1200 | 1 | 550 |
| RS5408 | GE | 19 | 1500 | 18 | 1400 | 18 | 1500 | 10 | 1200 | 15 | 1350 | 7 | 1100 | 1 | 1200 | 4 | 600 | 0 | 900 |
| RS5409 | GE | 5 | 1500 | 10 | 1500 | 5 | 1200 | 7 | 1299 | 9 | 1000 | 3 | 1000 | 0 | 600 | 1 | 800 | 1 | 750 |
| RS5410 | GE | 14 | 1000 | 12 | 700 | 11 | 600 | 1 | 700 | 2 | 500 | 16 | 500 | 6 | 900 | 3 | 900 | 13 | 1100 |
| RS5411 | GE | 40 | 1500 | 27 | 750 | 30 | 1500 | 16 | 1200 | 41 | 1300 | 8 | 750 | 5 | 1200 | 0 | 600 | 1 | 600 |
| RSA048 | GE | 15 | 700 | 19 | 800 | 19 | 800 | 3 | 600 | 2 | 800 | 25 | 700 | 1 | 500 | 6 | 400 | 0 | 600 |
| RSA056 | GE | 6 | 1000 | 9 | 1500 | 6 | 1200 | 9 | 1100 | 2 | 1000 | 7 | 1200 | 8 | 500 | 0 | 650 | 1 | 500 |
| RSA046 | GE | 0 | 700 | 3 | 600 | 13 | 700 | 0 | 400 | 0 | 400 | 0 | 600 | 0 | 600 | 0 | 650 | 3 | 700 |
| RSA049 | GE | 0 | 600 | 0 | 800 | 4 | 2000 | 1 | 600 | 1 | 600 | 4 | 750 | 2 | 400 | 0 | 850 | 3 | 1000 |
| RSA059 | GE | 2 | 500 | 0 | 600 | 0 | 650 | 0 | 400 | 4 | 600 | 1 | 550 | 0 | 500 | 0 | 800 | 0 | 900 |
| RS5407 | GE | 3 | 700 | 2 | 500 | 0 | 800 | 4 | 500 | 2 | 600 | 0 | 600 | 6 | 500 | 0 | 500 | 3 | 600 |
| RSA076 | NB | 0 | 1000 | 0 | 600 | 0 | 700 | 0 | 600 | 0 | 700 | 5 | 600 | 6 | 400 | 13 | 400 | 7 | 500 |
| RSB033 | NB | 8 | 700 | 0 | 500 | 8 | 600 | 10 | 650 | 13 | 500 | 8 | 500 | 19 | 400 | 8 | 400 | 5 | 700 |
| RSB034 | NB | 3 | 500 | 2 | 700 | 0 | 600 | 3 | 500 | 3 | 500 | 1 | 900 | 1 | 500 | 3 | 500 | 22 | 1200 |
| RSB035 | NB | 2 | 800 | 0 | 700 | 0 | 900 | 7 | 600 | 0 | 700 | 3 | 700 | 6 | 500 | 6 | 750 | 6 | 600 |
| RSB037 | NB | 0 | 600 | 4 | 1150 | 0 | 600 | 5 | 700 | 6 | 500 | 0 | 600 | 3 | 550 | 9 | 500 | 6 | 700 |
| RSC035 | NB | 0 | 550 | 3 | 850 | 2 | 700 | 0 | 800 | 2 | 500 | 2 | 700 | 1 | 500 | 5 | 600 | 5 | 600 |
| RSC036 | NB | 0 | 600 | 3 | 850 | 0 | 700 | 3 | 600 | 0 | 600 | 1 | 600 | 1 | 500 | 6 | 400 | 5 | 600 |
| RSC037 | PA | 3 | 900 | 4 | 750 | 10 | 700 | 0 | 500 | 2 | 500 | 1 | 650 | 9 | 600 | 2 | 800 | 1 | 650 |
| RSC046 | PA | 4 | 750 | 9 | 1050 | 3 | 700 | 1 | 800 | 1 | 550 | 3 | 700 | 1 | 500 | 0 | 700 | 1 | 650 |
| RSC047 | PA | 0 | 700 | 3 | 900 | 3 | 800 | 1 | 550 | 0 | 700 | 0 | 500 | 0 | 800 | 4 | 800 | 2 | 650 |
| RSC048 | PA | 1 | 1100 | 4 | 750 | 0 | 750 | 0 | 550 | 9 | 550 | 5 | 650 | 4 | 500 | 1 | 900 | 2 | 500 |
| RSC049 | PA | 5 | 1100 | 0 | 700 | 5 | 900 | 8 | 800 | 12 | 1100 | 7 | 800 | 1 | 600 | 2 | 600 | 2 | 500 |
| RSC050 | PA | 4 | 800 | 4 | 750 | 7 | 750 | 0 | 500 | 0 | 500 | 0 | 650 | 3 | 750 | 7 | 500 | 15 | 700 |
| RSB060 | PL | 2 | 700 | 15 | 900 | 3 | 800 | 0 | 500 | 4 | 650 | 1 | 550 | 0 | 600 | 0 | 700 | 0 | 600 |
| RSB066 | PL | 1 | 700 | 0 | 1300 | 0 | 900 | 1 | 850 | 0 | 600 | 1 | 500 | 0 | 200 | 0 | 400 | 3 | 800 |
| RSC051 | PL | 4 | 800 | 5 | 600 | 5 | 500 | 16 | 700 | 1 | 500 | 5 | 700 | 1 | 400 | 8 | 750 | 12 | 700 |
| RSC052 | PL | 3 | 800 | 3 | 800 | 4 | 900 | 2 | 1000 | 4 | 1200 | 15 | 1600 | 7 | 1100 | 0 | 900 | 4 | 700 |
| RSC053 | PL | 9 | 800 | 13 | 800 | 20 | 1200 | 18 | 800 | 27 | 1000 | 5 | 500 | 2 | 600 | 0 | 700 | 3 | 1000 |
| RSB059 | PL | 5 | 550 | 2 | 600 | 4 | 650 | 4 | 550 | 1 | 500 | 2 | 500 | 0 | 700 | 0 | 700 | 0 | 600 |
| RSB062 | PL | 2 | 600 | 4 | 750 | 3 | 700 | 2 | 900 | 1 | 850 | 0 | 600 | 10 | 750 | 14 | 1500 | 11 | 1550 |
| RSB064 | PL | 2 | 700 | 1 | 550 | 4 | 800 | 1 | 550 | 0 | 400 | 1 | 600 | 3 | 600 | 4 | 800 | 5 | 1050 |
| RSB065 | PL | 15 | 750 | 13 | 500 | 21 | 1500 | 9 | 700 | 8 | 650 | 11 | 1450 | 2 | 800 | 1 | 950 | 1 | 550 |
| RS5413 | SB | NA | NA | NA | NA | NA | NA | 8 | 550 | 0 | 500 | 3 | 500 | 0 | 400 | 0 | 500 | 2 | 650 |
| RS5412 | SB | 0 | 800 | 3 | 700 | 2 | 650 | 3 | 500 | 0 | 500 | 6 | 600 | 0 | 550 | 3 | 900 | 0 | 500 |
| RSB068 | SB | 3 | 400 | 0 | 600 | 1 | 500 | 0 | 500 | 3 | 900 | 3 | 1300 | 0 | 550 | 0 | 500 | 2 | 400 |
| RSB073 | SB | NA | NA | NA | NA | NA | NA | 2 | 600 | 8 | 600 | 0 | 500 | 3 | 1100 | 0 | 600 | 3 | 900 |
| RSB079 | SB | NA | NA | NA | NA | NA | NA | 1 | 550 | 0 | 600 | 5 | 600 | NA | NA | NA | NA | NA | NA |
| RSB069 | SB | 0 | 400 | 0 | 400 | 2 | 800 | 3 | 800 | 2 | 500 | 0 | 600 | 0 | 500 | 4 | 500 | 3 | 550 |
| RSB070 | SB | NA | NA | NA | NA | NA | NA | 4 | 750 | 2 | 500 | 2 | 650 | 3 | 550 | 12 | 700 | 16 | 600 |
| RSB071 | SB | NA | NA | NA | NA | NA | NA | 1 | 700 | 0 | 800 | 1 | 600 | 5 | 800 | 5 | 500 | 0 | 500 |
| RSB072 | SB | 3 | 500 | 8 | 700 | 18 | 800 | 3 | 600 | 6 | 600 | 5 | 550 | 2 | 600 | 1 | 500 | 0 | 700 |
| RSB074 | SB | 2 | 500 | 7 | 1500 | 23 | 1500 | 5 | 600 | 1 | 450 | 11 | 850 | 0 | 700 | 0 | 500 | 1 | 750 |
| RSB077 | SB | 0 | 400 | 1 | 500 | 1 | 300 | 10 | 700 | 9 | 900 | 7 | 750 | 6 | 600 | 3 | 500 | 4 | 750 |
| RSB080 | SB | 27 | 500 | 11 | 600 | 17 | 600 | 43 | 500 | 58 | 750 | 30 | 700 | NA | NA | NA | NA | NA | NA |
| RS5415 | SB | 13 | 1200 | 18 | 1100 | 14 | 1200 | 13 | 800 | 4 | 950 | 8 | 1100 | 27 | 1200 | 0 | 900 | 1 | 900 |
| RS5418 | SB | 5 | 1500 | 5 | 1500 | 4 | 1400 | 3 | 2000 | 0 | 1500 | 2 | 1600 | 2 | 1000 | 3 | 1000 | 0 | 700 |
| RS5419 | SB | 0 | 500 | 3 | 550 | 8 | 700 | 6 | 800 | 4 | 600 | 7 | 700 | 0 | 700 | 0 | 600 | 2 | 650 |
| RS5416 | SB | 9 | 400 | 0 | 500 | 1 | 550 | 3 | 750 | 3 | 550 | 3 | 600 | 1 | 650 | 1 | 500 | 3 | 500 |
| RS5417 | SB | 0 | 500 | 1 | 550 | 0 | 550 | 2 | 800 | 0 | 700 | 0 | 650 | 0 | 500 | 0 | 500 | 0 | 550 |
| RS5420 | SB | 0 | 400 | 0 | 500 | 3 | 500 | 0 | 600 | 0 | 700 | 0 | 800 | 0 | 500 | 0 | 500 | 0 | 1700 |
| RS5423 | SB | 0 | 500 | 0 | 400 | 0 | 400 | 0 | 700 | 1 | 650 | 4 | 800 | 1 | 500 | 0 | 500 | 1 | 650 |
| RS5421 | SB | 1 | 500 | 0 | 500 | 4 | 500 | 1 | 500 | 0 | 550 | 0 | 550 | 0 | 500 | 0 | 550 | 0 | 700 |
| RS5424 | SB | 0 | 450 | 0 | 500 | 0 | 650 | 0 | 700 | 1 | 650 | 0 | 650 | 9 | 1100 | 0 | 650 | 1 | 600 |
| RSB083 | SB | 0 | 500 | 0 | 600 | 0 | 500 | 1 | 650 | 1 | 400 | 0 | 550 | 0 | 500 | 0 | 500 | 2 | 600 |
| RSA096 | SS | 11 | 800 | 2 | 550 | 3 | 650 | 1 | 800 | 1 | 500 | 6 | 700 | 1 | 500 | 3 | 500 | 0 | 500 |
| RSA102 | SS | 5 | 500 | 6 | 700 | 3 | 700 | 2 | 800 | 4 | 500 | 1 | 700 | 0 | 500 | 0 | 500 | 2 | 500 |
| RSA106 | SS | 1 | 1100 | 2 | 1200 | 1 | 1200 | 1 | 1200 | 7 | 1500 | 5 | 1200 | 3 | 500 | 8 | 700 | 8 | 900 |
| RSA098 | SS | 4 | 1000 | 10 | 1800 | 2 | 1250 | 3 | 550 | 3 | 500 | 6 | 550 | 16 | 1700 | 14 | 1600 | 7 | 800 |
| RSA100 | SS | 13 | 1600 | 20 | 1800 | 6 | 1250 | 2 | 1550 | 6 | 1300 | 1 | 1400 | 5 | 550 | 0 | 1100 | 5 | 1500 |
| RSA104 | SS | 0 | 750 | 2 | 800 | 1 | 600 | 1 | 500 | 0 | 500 | 1 | 600 | 0 | 500 | 9 | 700 | 0 | 500 |
| RSA108 | SS | 9 | 650 | 13 | 1200 | 8 | 1350 | 0 | 1000 | 2 | 750 | 0 | 770 | 0 | 600 | 0 | 600 | 0 | 500 |
| RSA111 | SS | 4 | 500 | 9 | 800 | 11 | 700 | 2 | 700 | 1 | 900 | 2 | 700 | 0 | 700 | 4 | 700 | 0 | 650 |
| RSA097 | SS | 1 | 800 | 0 | 500 | 0 | 500 | 1 | 500 | 14 | 500 | 3 | 500 | 0 | 600 | 1 | 600 | 0 | 550 |
| RSA110 | SS | 6 | 500 | 8 | 700 | 5 | 700 | 0 | 500 | 2 | 550 | 0 | 500 | 0 | 600 | 0 | 500 | 0 | 750 |
| RSA069 | TB | 2 | 1700 | 0 | 1600 | 4 | 1100 | 0 | 750 | 0 | 850 | 0 | 550 | 0 | 600 | 0 | 500 | 0 | 750 |
| RSA071 | TB | 0 | 700 | 1 | 500 | 5 | 650 | 0 | 550 | 0 | 650 | 0 | 450 | 0 | 500 | 0 | 700 | 0 | 850 |
| RSA062 | TB | 4 | 850 | 5 | 1200 | 1 | 1600 | 1 | 1100 | 1 | 850 | 1 | 1000 | 13 | 1200 | 8 | 1400 | 13 | 900 |
| RSA064 | TB | 3 | 650 | 0 | 800 | 4 | 700 | 1 | 500 | 0 | 500 | 1 | 850 | 4 | 600 | 1 | 500 | 0 | 500 |
| RSA065 | TB | 26 | 600 | 16 | 1000 | 22 | 800 | 11 | 800 | 8 | 1000 | 10 | 750 | 1 | 500 | 7 | 600 | 0 | 500 |
| RSA066 | TB | 6 | 500 | 7 | 500 | 2 | 700 | 1 | 600 | 0 | 500 | 1 | 750 | 0 | 500 | 0 | 600 | 0 | 750 |
| RSA072 | TB | 1 | 600 | 0 | 650 | 1 | 600 | 0 | 550 | 0 | 500 | 0 | 750 | NA | NA | NA | NA | NA | NA |
| RSA073 | TB | 10 | 800 | 13 | 700 | 13 | 800 | 2 | 500 | 1 | 550 | 0 | 750 | 1 | 500 | 4 | 500 | 0 | 500 |
| RSB089 | TB | 1 | 1000 | 5 | 900 | 2 | 850 | 0 | 500 | 0 | 800 | 0 | 600 | 0 | 500 | 0 | 650 | 0 | 400 |
| RSB088 | TB | 3 | 750 | 10 | 800 | 6 | 700 | 3 | 550 | 0 | 600 | 1 | 500 | 0 | 750 | 0 | 550 | 0 | 600 |
| RS5402 | TB | 7 | 800 | 4 | 2000 | 6 | 1500 | 14 | 1000 | 7 | 550 | 17 | 600 | 0 | 600 | 6 | 900 | 0 | 750 |
| RS5405 | TB | 3 | 1400 | 2 | 1300 | 9 | 1500 | 1 | 1500 | 5 | 1100 | 1 | 1000 | 2 | 1200 | 1 | 1100 | 0 | 1000 |
| RSC093 | TB | 0 | 700 | 0 | 650 | 3 | 850 | 0 | 750 | 5 | 500 | 0 | 500 | 5 | 600 | 11 | 600 | 3 | 700 |
| RS5403 | TB | 9 | 950 | 11 | 850 | 9 | 650 | 1 | 750 | 3 | 700 | 1 | 650 | 1 | 800 | 0 | 800 | 0 | 750 |
| RSA089 | TB | 2 | 1250 | 21 | 1600 | 4 | 1800 | 6 | 1100 | 21 | 1600 | 2 | 1850 | 0 | 700 | 0 | 1050 | 1 | 1100 |
| RSA090 | TB | 0 | 1400 | 2 | 1400 | 0 | 850 | 0 | 1400 | 2 | 1400 | 0 | 850 | 7 | 650 | 3 | 500 | 9 | 500 |
| RSA091 | TB | 11 | 1600 | 23 | 1600 | 14 | 2100 | 5 | 900 | 9 | 900 | 4 | 750 | 4 | 800 | 11 | 1700 | 5 | 1200 |
| RSA092 | TB | 0 | 600 | 2 | 800 | 2 | 1100 | 0 | 1600 | 0 | 950 | 1 | 1700 | 0 | 1000 | 0 | 700 | 1 | 850 |
| RSB096 | TB | 13 | 700 | 6 | 1100 | 7 | 1000 | 0 | 750 | 0 | 700 | 0 | 600 | 2 | 650 | 5 | 600 | 6 | 900 |
| RS5404 | TB | 5 | 650 | 7 | 500 | 3 | 1000 | 0 | 700 | 1 | 550 | 0 | 500 | 3 | 900 | 2 | 700 | 1 | 500 |
| RS5334 | TB | 3 | 1200 | 5 | 1200 | 11 | 1500 | 1 | 1200 | 5 | 1300 | 2 | 1000 | 2 | 800 | 5 | 1000 | 0 | 900 |
| RS5336 | TB | 4 | 1300 | 4 | 1800 | 5 | 1500 | 5 | 900 | 10 | 1450 | 4 | 500 | 7 | 1600 | 5 | 1050 | 4 | 1000 |
| RS5337 | TB | 4 | 1100 | 7 | 1500 | 5 | 1100 | 2 | 1000 | 3 | 950 | 0 | 1100 | 2 | 650 | 9 | 1500 | 3 | 750 |
| RS5347 | TB | 4 | 1100 | 5 | 1600 | 3 | 1200 | NA | NA | NA | NA | NA | NA | NA | NA | NA | NA | NA | NA |
| RS5351 | TB | 17 | 1300 | 5 | 850 | 5 | 1600 | 1 | 750 | 9 | 1500 | 5 | 1700 | 0 | 900 | 0 | 900 | 4 | 1200 |
| RS5385 | TB | 0 | 1100 | 0 | 1100 | 2 | 1000 | 4 | 800 | 4 | 850 | 2 | 800 | 1 | 1100 | 0 | 1000 | 4 | 800 |
| RS5397 | TB | 14 | 900 | 3 | 1400 | 10 | 1400 | 5 | 1100 | 4 | 500 | 4 | 600 | 9 | 600 | 35 | 850 | 15 | 1000 |
| RS5399 | TB | 4 | 1500 | 2 | 900 | 3 | 1000 | 1 | 1200 | 1 | 800 | 3 | 900 | NA | NA | NA | NA | NA | NA |
| RSA113 | TB | 16 | 1200 | 14 | 1350 | 21 | 1050 | NA | NA | NA | NA | NA | NA | NA | NA | NA | NA | NA | NA |
| RSB120 | TB | 2 | 800 | 3 | 700 | 1 | 1000 | 4 | 800 | 3 | 650 | 6 | 1000 | 0 | 600 | 2 | 650 | 1 | 550 |
| RSC094 | TK | 0 | 1000 | 5 | 650 | 1 | 650 | 1 | 500 | 0 | 650 | 2 | 750 | 1 | 400 | 0 | 500 | 2 | 600 |
| RSC095 | TK | 6 | 850 | 7 | 950 | 2 | 950 | 0 | 500 | 4 | 550 | 0 | 600 | 0 | 400 | 2 | 400 | 1 | 650 |
| RSC096 | TK | 7 | 950 | 3 | 750 | 4 | 850 | 0 | 400 | 2 | 500 | 0 | 750 | 4 | 400 | 0 | 500 | 0 | 500 |
| RSC097 | TK | 0 | 500 | 0 | 600 | 0 | 500 | 4 | 550 | 5 | 700 | 1 | 550 | 0 | 700 | 2 | 800 | 1 | 650 |
| RSC098 | TK | 1 | 600 | 0 | 550 | 7 | 700 | 1 | 600 | 0 | 600 | 0 | 600 | 2 | 500 | 0 | 600 | 1 | 800 |
| RSC099 | TK | 9 | 650 | 21 | 600 | 28 | 850 | 2 | 850 | 4 | 550 | 19 | 500 | 10 | 600 | 8 | 800 | 6 | 850 |
